# Benign Metastasizing Leiomyomatosis to the Skin and Lungs, Intravenous Leiomyomatosis, and Leiomyomatosis Peritonealis Disseminata: A Series of Five Cases

**DOI:** 10.1093/oncolo/oyab019

**Published:** 2022-01-28

**Authors:** João Boavida Ferreira, Rafael Cabrera, Filipa Santos, Andreia Relva, Hugo Vasques, António Gomes, António Guimarães, António Moreira

**Affiliations:** Serviço de Oncologia Médica, Instituto Português de Oncologia de Lisboa Francisco Gentil, Lisbon, Portugal; Serviço de Anatomia Patológica, Instituto Português de Oncologia de Lisboa Francisco Gentil, Lisbon, Portugal; Serviço de Anatomia Patológica, Instituto Português de Oncologia de Lisboa Francisco Gentil, Lisbon, Portugal; Serviço de Ginecologia, Instituto Português de Oncologia de Lisboa Francisco Gentil, Lisbon, Portugal; Serviço de Cirurgia Geral, Instituto Português de Oncologia de Lisboa Francisco Gentil, Lisbon, Portugal; Serviço de Ginecologia, Instituto Português de Oncologia de Lisboa Francisco Gentil, Lisbon, Portugal; Serviço de Oncologia Médica, Instituto Português de Oncologia de Lisboa Francisco Gentil, Lisbon, Portugal; Serviço de Oncologia Médica, Instituto Português de Oncologia de Lisboa Francisco Gentil, Lisbon, Portugal

**Keywords:** benign metastasizing leiomyomatosis, intravenous leiomyomatosis, *leiomyomatosis peritonealis disseminata*, hysterectomy, lung nodules

## Abstract

Benign metastasizing leiomyomatosis (BML) is a rare disease that typically occurs in women with a history of uterine leiomyomatosis. Benign metastasizing leiomyomatosis occurs more frequently in the lungs but may also develop in other organs and tissues. Other unusual variants of extra-uterine leiomyomatosis include intravenous leiomyomatosis (IVL) and leiomyomatosis peritonealis disseminata (LPD). In this article, three cases of BML are presented. One case, in a premenopausal woman, presented cutaneous metastases. We also present a case of IVL and a case of LPD, which occurred in postmenopausal women. Given the rarity of BML, IVL, and LPD, the authors reviewed the literature and herein discuss the implications for treatment in all five cases. Evidence for treating BML, IVL, and LPD is still scarce, and data available from our series and other small series seem to point to the patient’s hormonal status playing a fundamental part in the treatment plan. Furthermore, a collecting bag when performing excision of uterine leiomyomas may help avoid the potential spreading of leiomyomatosis. Hysterectomized patients with chronic cough, frequent respiratory infections, abdominal discomfort, right heart failure, or non-specific symptoms should be actively screened for BML, IVL, and LPD. Treatment should be individualized according to each patient’s hormonal status and desires.

## Teaser

Benign metastasizing leiomyomatosis is a rare disease that typically occurs in women with a history of uterine leiomyomatosis. Other unusual variants of extra-uterine leiomyomatosis include intravenous leiomyomatosis and leiomyomatosis peritonealis disseminata. This article presents a series of five cases.

## Introduction

Benign metastasizing neoplasias are a group of neoplasias that, despite the absence of anaplasia, have the capacity to metastasize. Examples include meningioma,^1^ uterine leiomyoma,^2^ giant cell tumor of bone,^3^ salivary glands, and nevi cell aggregates in cervical lymph nodes.^4,5^ Benign metastasizing leiomyomatosis (BML) is a rare disease that typically occurs in women with a history of uterine leiomyomatosis. A little over 100 cases have been described,^6-10^ although the frequency of reporting seems to be rising, purportedly due to increased awareness and knowledge of the disease. Uterine leiomyomas are the most common benign gynecological neoplasms in women, affecting around 30% of women over 35 years old.^6,11,12^ Leiomyomatosis usually affects women of childbearing age.^6,7^ Benign metastasizing leiomyomatosis consists of multiple metastatic nodes formed by smooth muscle cells.^2,13^ The first mention of BML might have been made by Krische,^14^ with the first case with a histological description being reported by Steiner,^2^ in a 36-year-old woman with multiple uterine leiomyomas, multiple solid and cystic pulmonary leiomyomas and 1 tracheobronchial lymph node with a leiomyomatose metastasis. Awonuga et al.^15^ suggested that metastization may happen via lymphatic dissemination or by in situ hormone-sensitive proliferation of smooth muscle cells. According to a systematic review by Barnas et al.,^16^ only a minority of cases of BML develop without a history of gynecological surgery (<20%), supporting the hypothesis of iatrogenic lymphatic dissemination. Another hypothesis put forward to help explain BML is peritoneal seeding after myomectomy, or hysterectomy via laparotomy or laparoscopic morcellation of the uterus.^17^

Benign metastasizing leiomyomatosis occurs more frequently in the lungs^16^ but may also develop in other organs and tissues (eg, heart, spinal cord (potentially causing spinal cord compression)^7,18,19^). Bones can also be affected by BML.^20^ Intravenous leiomyomatosis occurs in 0.25% to 0.40% of patients with uterine leiomyomas.^19,21,22^ From these, IVL reaches the heart in 10% to 40% of cases.^6,7,23^ Recently, Wolfe et al.^24^ described a case of perivascular leiomyomatosis in a patient with multiple leiomyomatose nodules within the tunica media, tunica adventicia and tunica adventicia-related adipose tissue surrounding the saphenous vein and adjacent venous structures.

Intravenous leiomyomatosis (IVL) was first described by Birch-Hirschfield.^25^ There are around 200 to 300 cases of IVL reported in the literature.^22,26-28^ A commonly accepted hypothesis for the origin of IVL is hematogenous dissemination from a uterine leiomyoma.^6,7,24,27,29,30^ It is not yet clear whether IVL originates from uterine smooth muscle or from venae uterinae smooth muscle.^31-36^ Intravenous dissemination can spread more often via a uterine vein, less often via the ovarian vein.^23^ From the uterine vein, it can spread into the internal iliac veins and then the inferior vena cava. From the ovarian vein, it can spread directly into the inferior vena cava on the right side and via left renal vein on the left side. Ultimately, IVL may reach the right atrium (intracardiac leiomyomatosis), and even progress to the pulmonary arteries.^23^ The first case of intracardiac IVL was described by Dürck.^37^ It can also be associated with lung nodules (pulmonary BML).^28,30^ Symptoms of pelvic IVL are similar to those caused by uterine leiomyomas.^26^ Pelvic IVL may cause menometrorrhagia and pelvic discomfort.^23,29^ Diagnosis is often made by chance during microscopical analysis of the uterus after hysterectomy.^26^ Extrapelvic IVL, especially in the inferior vena cava, causes a congestive heart failure-like syndrome, with abnormality of venous return and eventually chest pain, peripheral edema, syncope, and pulmonary embolism.^7,23,29^ In case of intracardiac leiomyomatosis, obstruction of the tricuspid orifice may lead to cardiac arrest and sudden death.^6,23,29^ The diagnosis of intracardiac IVL must be suspected in fertile women with a history of past or present uterine leiomyomatosis with a mobile mass in the right atrium and inferior vena cava, without invading the endothelium nor the endocardium, seen on an echocardiogram.^23^ The intracardiac IVL tumor appears as a snaking, free, moving mass inside the vases, and the heart.^7,29,30^

Only a little over 200 cases of *leiomyomatosis peritonealis disseminata* (LPD) are described in the literature.^38^ The fact that it can appear in men^39-42^ and in women who have not had a prior uterine leiomyoma and/or who are postmenopausal might imply an extra-uterine origin for the development of the condition in these groups, through uterine metaplasia.^6,29,38^*Leiomyomatosis peritonealis disseminata* was first described by Willson and Peale.^43^ Usually, LPD occurs in fertile women,^38,44^ and it seems to be associated with having uterine leiomyomatosis, operated or not.^38^ Interestingly, a different type of extra-uterine leiomyoma (yet still originating from the uterus) has been described: parasitic leiomyoma. It has been postulated that a parasitic leiomyoma is generated by a twist in a subserosal uterine leiomyoma that consequentially loses its attachment to the uterus, thence becoming independent within the abdominal cavity. A major risk factor for developing LPD might be peritoneal seeding after morcellation hysterectomies or myomectomies with subsequent abdominal spreading of leiomyoma fragments; this is even more important when leiomyosarcoma is suspected, so a collecting bag to be used with morcellation is recommended.^45^ Another risk factor to be considered is embolization of the uterine artery for treatment of a uterine leiomyoma.^43^ The majority of patients with LPD are asymptomatic.^29,38,46^ When present, symptoms may include abdominal discomfort, rectal bleeding, metrorrhagia, abdominal distension, or an abdominal mass.^38,46^ Ascitis and/or multiple lymphadenopathies may also be involved in LPD.^46^ Compression of surrounding tissues by LPD may underlie ureteral obstruction, renal colic, pyelonephritis, and acute renal injury.^38^ Main differential diagnosis includes peritoneal leiomyosarcomatosis and peritoneal carcinomatosis. Unlike peritoneal leiomyosarcomatosis, peritoneal nodules in LPD are well circumscribed.^6^ On MRI, the T2 signal of LPD nodules is low, whereas that of peritoneal carcinomatosis is high.^44^

Uterine leiomyomatosis is also associated with cutaneous leiomyomas and renal cell carcinoma in the hereditary leiomyomatosis and renal cell cancer (HLRCC) syndrome an autosomal dominant disorder with a mutation in the tumor suppressor gene coding for fumarate hydratase (fumarase).^47^ Two hundred families are known in the literature.^47^ Other organs may also develop tumors in this syndrome.^26^

Benign metastasizing leiomyomatosis smooth muscle cells express estrogen and progesterone receptors on immunohistochemical staining, but are usually negative for Ki-67, which means that the metastatic nodes have a low proliferative index.^48^ Immunohistochemical staining helps differentiate BML from leiomyosarcoma (BML has a low Ki-67 proliferative index^48-50^). Terminal deletions in chromosomes 19q and 22q have been found to be associated to BML.^49,51^ This cytogenetic profile is not shared with pulmonary chondroid hamartomas, which tend to have rearrangements of 12q15 and 6p21, but not 19q and 22q deletions,^51^ nor with leiomyosarcomas, which tend to have more complex karyotypes (extra chromosomes, complex rearrangements, and marker chromosomes^52^). t(12;14) and del(7q) have been reported as the most common cytogenetic rearrangements in uterine leiomyomatosis.^53-56^ In a study of more than 800 karyotyped uterine leiomyomata, a subgroup of around 1% had deletions in chromosome 1, frequently with monosomies 19 and/or 22.^57^ It was also found that in the 1p deletion subgroup, the transcriptional profile resembled that of leiomyosarcoma, suggesting that there might be a common pathogenic pathway.^51,57^ 1p, 19q, and 22q might therefore be used as markers for uterine leiomyomata with the potential to metastasize, especially to the lungs.^49,51^ Their relevance might be highest in the context of de novo lung nodes in women with a history of uterine leiomyomata,^49^ especially if a uterine sample is not available.^49^ Due to the rarity of BML, actively performing a genetic screening in women undergoing surgery due to uterine leiomyomatosis is not recommended.^49^ Changes in chromosomes 1 and 22 have also been reported in IVL.^58^ In a series of 28 IVL cases, the most common changes occurred, in order of decreasing frequency, in chromosomes 1p, 22q, 2q, 1q, 13q, and 14q. Interestingly, in this series, the 28 IVL cases were clustered in three genomic groups, which were comparable to molecular subtypes in uterine leiomyomatosis.^58^ A series of eight cases of LPD^59^ found an array of chromosomal changes, including 12q changes, del(14q), del(22), ins(8), del(7), t(3;11), and add(11q). An other series of eight cases^60^ found through immunohistochemistry that all uterine and extra-uterine tumors were positive for *HMGA2* (over-expression) and *MED12* (either mutation or low expression), both in uterine and extra-uterine leiomyomas. In this series, only two out of eight patients had not been submitted to morcellation of uterine leiomyomas. *MED12* is reported to be the most frequently altered gene in uterine leiomyomas,^61^ and changes in either *MED12* or *HMGA2* are present in 80%-90% of uterine leiomyomas.^62^

Benign metastasizing leiomyomatosis may present as asymptomatic lung incidentalomas, or as lung nodes synchronous with uterine leiomyomas or posthysterectomy.^9^ The latter corresponds to most cases, and in these cases diagnosis is made fortuitously by a chest X-ray.^6,9,29^ Progression in number and size of lung nodes may lead to symptoms of chronic cough,^57^ wheezing,^7^ shortness of breath,^9^ flu-like symptoms,^51^ chest pain,^63^ hemoptysis,^64^ right heart failure with peripheral edema, ascites and hepatomegaly due to obstruction of the blood flow through the lungs,^2^ clubbed fingers,^2^ cyanosis,^2^ progressive respiratory failure,^65^ pulmonary embolism,^28^ and potentially death.^2,65^

Benign metastasizing leiomyomatosis usually appears in imaging exams as solitary or multiple interstitial nodes that do not enhance with intravenous contrast, and that also do not tend to involve the endobronchial or pleural spaces.^6^ Bilateral involvement is seen in 70% of cases.^7,16,28,48^ Rare presentations include miliary BML,^66^ cavitary lung nodules, interstitial lung disease, and multiloculated fluid-filled cysts.^67^

Benign metastasizing leiomyomatosis has also been linked to lymphangioleiomyomatosis (LAM). Both diseases tend to occur in fertile women and to involve the proliferation of smooth muscle cells. LAM most commonly affects the lungs^68^ and seems to worsen with menses,^69^ pregnancy,^70-72^ and the use of oral contraceptives.^73^ Interestingly, uterine leiomyomas have a higher mitotic count during the secretory phase of the menstrual cycle, being even higher in younger women.^74^ Also, estrogen and progesterone are major promoters of uterine leiomyoma growth.^75^

We report three cases of BML to the skin and lungs, 4, 16, and 22 years after histerectomy, in premenopausal women, two of them diagnosed accidentally after a chest X-ray (one patient had a chest X-ray performed due to symptoms of a respiratory infection, and the other was asymptomatic and had a routine chest X-ray). The three cases had different treatment approaches, one surgical, one medical, and one medical + surgical. We also report one case of IVL in a nonhysterectomized, postmenopausal woman, and one case of LPD in a hysterectomized, postmenopausal (surgically induced) patient.

## Case 1

Premenopausal woman, menarche at the age of 12, 0 deliveries, hysterectomy for hemorrhagic uterine leiomyomata at the age of 23. The patient has Gilbert syndrome and no other medical history. The patient also did not have a family history of any cancer or tumors, including renal cell cancer. Sixteen years after hysterectomy (at the age of 39), multiple lung nodules were found on a chest X-ray performed during workup of an upper respiratory infection. A chest CT scan revealed multiple lung nodules, 4-5 cm in diameter. Abdominal and pelvic CT scan showed no changes. A right upper lobe lung resection was performed via right videothoracoscopy. Pathology reported a lung fragment with various small, well-circumscribed nodules dispersed in the lung parenchyma. These nodules consisted of mesenchymal fascicular neoplasms composed of spindle cells with eosinophilic cytoplasm and cigar-shaped nuclei with inconspicuous nucleoli. There was mild cytological atypia and no mitotic figures. No necrosis or high-grade features were observed. It is not known whether the patient was started on hormone therapy. Circa 6 months after surgery, the patient developed dyspnea on exertion. A chest CT scan was repeated, showing a rise in number and size of the lung nodules. Blood tests had no change in levels of FSH, LH, and estradiol. Bilateral annexectomy was therefore performed, with ensuing menopause. The right ovary had an endometrioid cyst, and there were no other changes. It is not known whether any medication was started at this phase. Six years on (as the patient was 46 years old), new nodules appeared on the forearms, anterior thoracic wall and on the back. Biopsy of the cutaneous nodules confirmed leiomyomatosis. Since then the disease has remained stable and no medication was started. Follow-up is being made annually with a chest CT scan. In our revision of this patient’s case, we stained the lung nodules samples to look for fumarate hydratase expression and found that the tissue in the lung samples lacks expression of fumarate hydratase. The patient has no personal or family history of cancer or of leiomyomatosis. We referred the patient to our Family Risk Clinic given the possibility of HLRCC syndrome. Unfortunately, we did not have access to skin nodes samples or to the uterine leiomyomata tissue, so we could not perform a clonality analysis.

## Case 2

**Figure 1.**
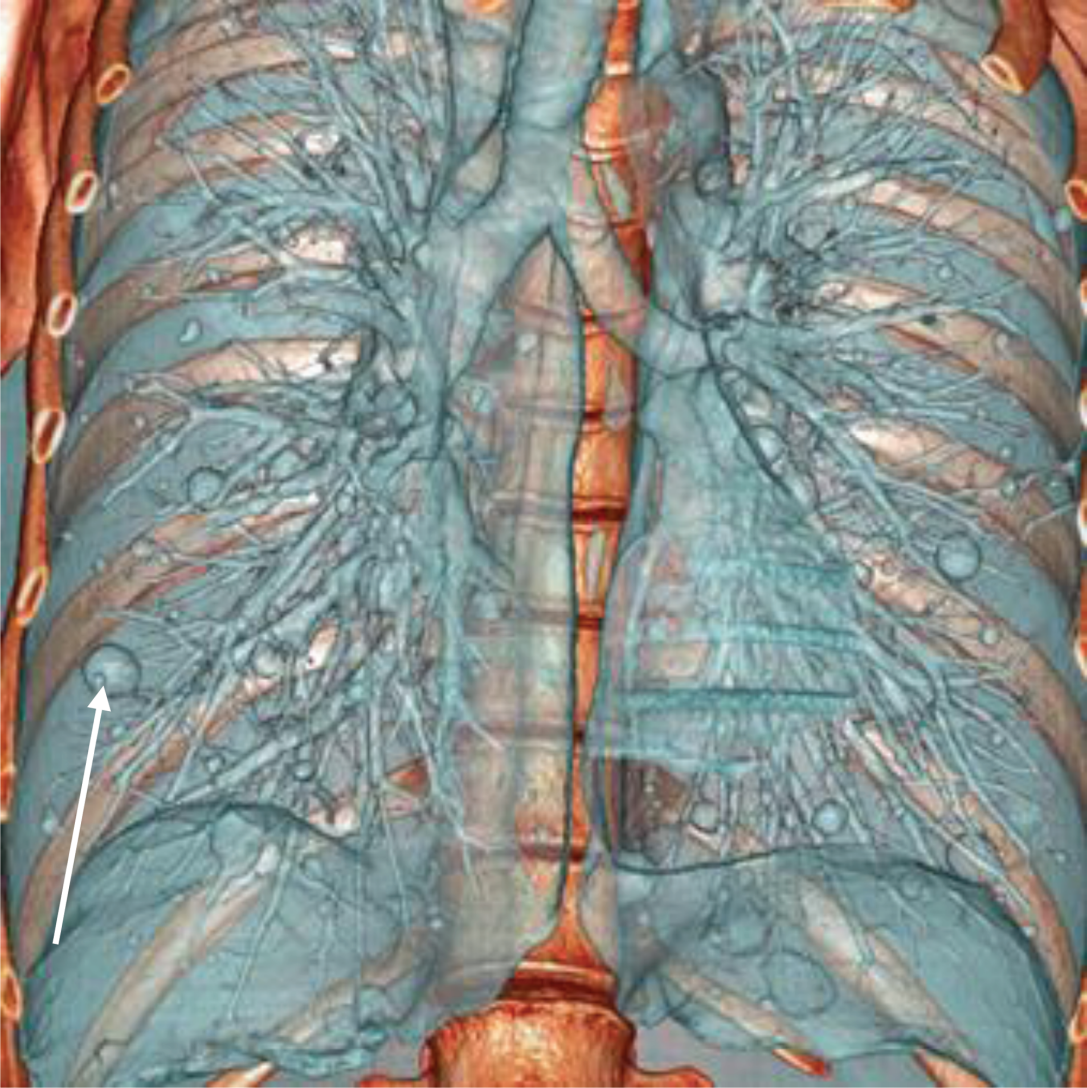
3D reconstruction of a chest CT scan, anterior view. Lung nodules in a snowfall pattern in the context of pulmonary benign metastasizing leiomyomatosis in Patient 2. The patient was started on goserelin, 3.6 mg subcutaneous every 28 days. The disease has remained stable, with the lung nodules reaching no more than 13 mm in diameter. The white arrow is pointing to one of the nodules, in the right lung.

Premenopausal woman, menarche at the age of 13, one dystocic delivery by cesarean section, with a history of hysterectomy for hemorrhagic uterine leiomyomata at the age of 23, and no other past diseases. The patient had a sister who had her menopause at the age of 52 and another sister who had a late menopause at the age of 56. Twenty-two years after hysterectomy (at the age of 45), multiple lung nodules were found on a routine chest X-ray. On examination the patient had clubbed fingers. A chest CT scan was performed, revealing multiple, scattered lung nodules, and maximum 10-15 mm in diameter. A lung biopsy containing three intraparenchymal lung nodules was obtained, showing three nodules of fibromuscular tissue, with no atypia or mitoses. Pathology report was compatible with BML (with the appearance of “fibroleiomyomatose hamartomas”). An endovaginal and suprapubic ultrasound scan revealed no pelvic leiomyomas. Blood tests had no change in levels of FSH, LH, and estradiol. The patient was started on hormonal blocking agents with the gonadotropin-releasing hormone (GnRH) agonist goserelin, 3.6 mg as a subcutaneous implant every 28 days, and since then no attempt has been made to discontinue treatment. Due to this, there has been no assessment of the patient’s hormone status. The disease has remained stable with the lung nodules maintaining a maximum diameter of 13 mm, and follow-up is being made annually with a chest CT scan. A follow-up chest CT scan is shown in Figure 1.

## Case 3

Premenopausal woman, menarche at the age of 11, one high-risk pregnancy due to placental abruption and rupture, one spontaneous abortion due to fetal growth restriction and a third pregnancy without complications, two cesarean sections, with a history of hysterectomy for a hemorrhagic uterine leiomyoma complicated by retroperitoneal bleeding with acute anemia at the age of 40. The patient is obese (body mass index, BMI: 30.1 kg/m^2^), has a history of dyslipidemia, smoking in late adolescence (7 pack-years), childhood adenoidectomy, childhood asthma and vertebral bone degenerative disease, and an allergy to etoricoxib (facial edema). The patient has no personal history of cancer but has a family history of cancer: father died at 57 with oral cavity and esophageal cancers (was a smoker and alcoholic), paternal grandfather died at 72 with lung cancer (was a smoker), paternal uncle died at 50+ with pancreatic cancer, maternal uncle died at 50 with gastric cancer. There was no history of renal cell cancer or of other tumors in the family. Four years after hysterectomy (at the age of 44), the patient developed what seemed to be a recurrence of her childhood asthma symptoms, with episodes of dyspnea, wheezing, fatigue, and hypersomnia. The symptoms would exacerbate on physical exertion. A chest X-ray revealed a right lung mass. A subsequent thoraco-abdominopelvic CT scan showed a tissue-dense, spiked-countour node on the anterior segment of the right upper lobe, invading the mediastinal pleura, plus a second tissue-dense, bilobed, ampulliform mass in the left pulmonary hilum, involving the left pulmonary artery anteriorly and compressing the left main bronchus and adjacent segmental bronchii. There were no lymphadenopathies or any other findings. A PET scan revealed increased metabolism of the lung lesions. The right lung node was biopsied, presenting a fusocellular proliferation, without atypia, necrosis or mitotic activity, positive for desmin, caldesmon, estrogen receptors, and actin (weak expression). Remaining markers were negative, namely cytokeratins, ALK1, S100, CD34, myogenin, myoD1, inhibin, calretinin, CD117, and HMB-45. This result was compatible with a benign mesenchymal neoplasia with smooth muscle differentiation. A fine needle aspiration cytology was performed on the left lung hilar mass, which showed the same fusocellular, benign proliferation pattern. CA 125 and routine blood tests were normal. Serum assessment of estradiol and FSH and LH were at premenopausal levels. The patient was started on hormone therapy with goserelin 3.6 mg every 28 days subcutaneous + letrozole 2.5 mg once daily. Estrogen levels decreased from premenopausal levels of 166 to <9 pg/mL. The patient mentioned no desire to have more children and was submitted to bilateral annexectomy. After bilateral annexectomy, goserelin was discontinued and letrozole was kept at 2.5 mg once daily. At a follow-up assessment, serum hormone levels were found to be compatible with postmenopausal levels. A follow-up chest CT scan performed 6 months after starting hormonal blockade and 2 months after surgical castration showed a significant reduction of the pulmonary lesions’ size, the right upper lobe mass with a 74% reduction in size, and the left pulmonary hilum mass with a 44% reduction in size. The patient has been on respiratory kinesitherapy and her symptoms have improved.

## Case 4

**Figure 2.**
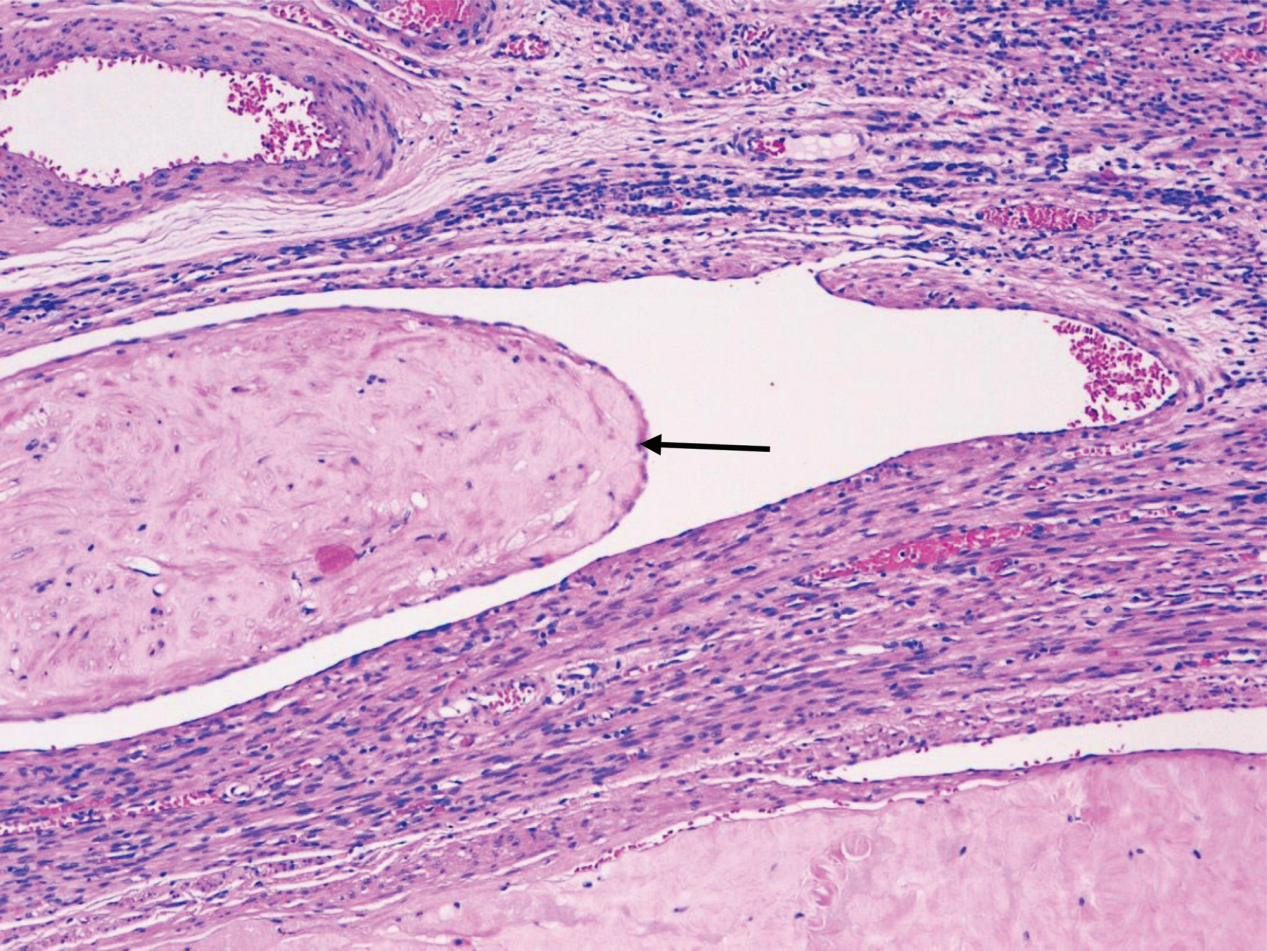
Microscopic appearance of intravenous leiomyomatosis in Patient 4 (H&E stain). An intravascular leiomyoma can be seen left of the center in the image. This leiomyoma has been re-epithelized with vascular endothelium (black arrow).

Postmenopausal woman, menarche at the age of 13, without exposure to hormone replacement therapy, gesta 3 with three eutocic deliveries, who developed a progressively worse abdominal pain over the course of 4 months, at the age of 61. No vaginal bleeding. The patient suffered from hypertension and diabetes mellitus. An abdominal and pelvic MRI showed an enlarged uterus due to a heterogeneous tumor with 10.9 cm at its longest axis, with an intracavitary component and an extrauterine component extending for 18.4 cm down to the Douglas pouch; multiple subserous leiomyomas were also described. Staging exams did not show any metastases. Hormonal levels (FSH, LH, and estradiol) are unknown. The patient was submitted to hysterectomy, a bilateral annexectomy and a bilateral lymphadenectomy. Pathology revealed multiple subserous nodules corresponding to uterine leiomyomatosis and the bulging mass corresponding to IVL (Figure 2). The right ovary was partially occupied by a solid, well circumscribed, white nodule measuring 3.8 cm. In the hysterectomy specimen, the myometrium was distended by various well-circumscribed, fascicular, white nodules, some of them with worm-like features. Also, a similar solid, well-demarcated nodule with 5.2 × 5 × 3 cm was found in the right parametrium. Histologically, all nodules were composed of fascicules of spindle cells, with eosinophilic cytoplasm and elongated nuclei, with focal nucleoli. Some of these nodules observed in the myometrium and the parametrium had intravascular growth. No necrosis or cytological atypia were found and an inconspicuous mitotic rate was present. It is not known whether the patient was started on hormone therapy. The patient was discharged and had her further follow-up with her general practitioner.

## Case 5

Woman, menarche at the age of 11, one pregnancy that ended in abortion, with a history of hysterectomy and a bilateral annexectomy (with consequent menopause) for a large, hemorrhagic uterine leiomyoma (20 × 16 × 6 cm) at the age of 24. The patient is obese and smokes. The patient has no personal history of cancer but has had a paternal grandaunt who had a cancer diagnosis which the patient could not specify. No other tumors were reported in the family. Five years after hysterectomy (at the age of 29), the patient developed abdominal pain and distension, and a very large pelvic tumor with 25 cm at its longest axis was found, with at least three additional tumors between the former and the anterior abdominal wall (the biggest two with 9.6 and 6.8 cm). Laparotomy was performed and the larger tumor was excised. Multiple other abdominal nodules were also found. Pathology of the pelvic tumor revealed a uterine leiomyoma (actin+ and desmin+, without atypia or necrosis). Three months later the patient developed bilateral hydronephrosis due to compression of both ureters, and had stents placed. One week later a retroperitoneal dissection was performed and all the visible nodules were removed. Pathology of the nodules confirmed the diagnosis of leiomyomata (well circumscribed, fascicular nodules composed of spindle cells with eosinophilic cytoplasm and cigar-shaped nuclei with inconspicuous nucleoli, mild cytological atypia and no mitotic figures, no necrosis or high-grade features were observed). It is not known whether the patient was started on hormone therapy at this stage. One year later (as the patient was 30) a control CT scan showed new nodules in the abdominal wall. In the following year, when the patient was 31, a control CT scan showed multiple nodular images compatible with fibromas already in the peritoneum and also in the abdominal wall. A biopsy confirmed LPD once again. Hormonal levels (FSH, LH, and estradiol) were low, compatible with postmenopause. GnRH agonist goserelin was started, but in January 2011 a new CT scan showed disease progression. Goserelin was stopped and letrozole was started. In the many CT scans performed so far, numerous enlarged periaortic and iliac lymph nodes were visible, but none was ever biopsied. The patient remained asymptomatic, with control CT scans showing stable disease, and at the age of 35 a tumor appeared in the right iliac fossa. This mass was removed and the tumor pathology exam confirmed a new recurrence of extra-uterine leiomyomatosis. Control CT scans were repeated annually. A control CT scan showed progression of the LPD (biggest nodule 42 mm in diameter) and the abdominal wall leiomyomata (biggest nodule 41 mm in diameter); the vaginal pouch did not show any changes. Several visceral leiomyomas were also found. Again, in this CT scan the existence of multiple enlarged periaortic and iliac lymph nodes was reported. Concurrently multiple unsuspicious enlarged mediastinal lymph nodes were found anew. Lymph nodes were not biopsied. The patient has remained asymptomatic ever since. Control CT scans never revealed any nodules outside the abdomen, namely in the lungs.

## Discussion

Given the rarity of BML, there is no standard treatment or guidelines. Differential diagnosis is important to exclude other causes of lung nodules. A family history should be taken in order to exclude HLRCC.

Differential diagnosis of pulmonary BML includes non-infectious and infectious granulomatous diseases, lung smooth muscle diseases, autoimmune diseases, and other diseases that affect the lungs’ interstitium. A proposed differential diagnosis is presented in Table 1

**Table 1.** Proposed differential diagnosis of pulmonary benign metastasizing leiomyomatosis.

Infectious granulomatous diseases	Tuberculosis
	Non-tuberculous mycobacterioses
	Fungal lung infection
	Syphilis
	Tularemia
	Cat-scratch disease
	Whipple disease
	Parasitoses
Non-infectious granulomatous diseases	Sarcoidosis
	Inflammatory bowel disease
	Pneumoconioses
	Hypersensitivity pneumonitis
	Drugs
	Foreign body reaction
Lung smooth muscle diseases	Leiomyomatosis
	Pulmonary hamartomas
	Lymphangioleiomyomatosis
	Leiomyosarcoma
Autoimmune diseases	Vasculitis
	Rheumatoid arthritis
Other diseases	Langerhans cell histiocytosis
	Granulomatous lymphocytic interstitial lung disease
	Alport syndrome (esophageal, tracheobronchial, and vulval leiomyomatosis with nephropathy)

It is important to distinguish IVL from other leiomyomatoses. Given the reports in the literature, IVL can occur as an extension of uterine leiomyomatosis. However, other uterine tumors may invade the intravenous space, most notably uterine sarcomas (intravenous sarcomatosis uteri).^76^ The main differential diagnosis of intracardiac IVL is made with cardiac myxoma, a specifically intracardiac tumor generally occurring in the left atrium, without inferior vena cava involvement, and adhering to the endocardium mainly through the interatrial septum.^48^ Other differential diagnoses include thrombi, cardiac metastases, and cardiac leiomyosarcoma, the latter two with solid masses adherent to the heart wall.^23^

In the systematic review by Barnas et al.,^16^ the average time from uterine surgery to BML diagnosis was found to be 8.8 years. A report of 10 cases of BML found an interval of 4 to 23 years, with a median of 16 years.^77^ In a literature review by Jautzke et al.,^13^ 74 reported cases of BML yielded an interval of 3 to 20 years, with an average of 10 years. Miller et al.^50^ (10 cases) reported a median time of 18.5 years. Barnas et al.,^16^ in their review of 161 cases, report a mean age of 47.3 years at diagnosis of BML. Most symptomatic metastases were detected, which does not rule out the presence of micro/subclinical metastases before. In the series of 10 cases by Miller et al.,^50^ the median age at diagnosis was 55 years. As for IVL, most patients are diagnosed when they are premenopausal and between 40 and 50 years old.^21,22,29^ Cases of LPD are found around the age of 36 on average.^29,38^ We compared our BML cases with the review by Barnas et al.,^16^ and the series published by Jautzke et al.,^13^ Miller et al.,^50^ and Kayser et al.,^77^ in Table 2.

**Table 2.** Comparison of age at hysterectomy/myoma surgery, age at diagnosis of BML, median time from uterine surgery to BML diagnosis, and patient hormonal status at the time of diagnosis of BML, between our three cases of BML and published series.

Cases	Age at hysterectomy/myoma surgery(years)	Age at BML diagnosis (years)	Time between hysterectomy/myoma surgery and BML diagnosis (years)	Hormonal status at BML diagnosis
Barnas et al.^16^^a^ (161 cases) (review of literature)	Mean 38.5 Min. 18 Max. 72	Mean 47.3 Min. 22 Max. 77	Mean 8.8 Min. 0 Max. 31	Predominantly PERI
Jautzke et al.^13^ (5 cases)	Median 45 Min. 34 Max. 48	Median 51 Min. 36 Max. 54	Median 3 Min. 0 Max. 12	PRE-PERI
Kayser et al. ^77^ (10 cases)	Median 34.5 Min. 23 Max. 45	Median 47.5 Min. 40 Max. 66	Median 16 Min. 3 Max. 23	Predominantly PERI
Miller et al. ^50^ (10 cases)	Median 35 Min. 24 Max. 58	Median 55 Min. 42 Max. 71	Median 18.5 Min. 0 Max. 36	PERI-POST
Case 1	23	39	16	PRE
Case 2	23	45	22	PRE
Case 3	40	44	4	PRE

The data provided by this paper included the values for the mean, not for the median, so the mean values were introduced in the table.

Abbreviations: PERI, perimenopausal; POST, postmenopausal; PRE, premenopausal.

Benign metastasizing leiomyomatosis generally has an indolent evolution,^26^ and a watch-and-wait strategy might be an option. This strategy is in line with several reports of indolent cases of BML, with spontaneous regression having been described.^6,8,10,11,50^ In case of progression, complications or symptoms, a therapeutic approach must be contemplated. Historically, oophorectomy has been used as a first line option in the treatment of BML. By surgically castrating the patients, a low-estrogen environment can be maintained. Surgical castration, apart from causing iatrogenic menopause in otherwise fertile women, puts doctors and patients in a quandary in cases where the patient still wishes to remain fertile or when surgery is not possible.^78^ In addition to this, the results obtained with hormone therapy (GnRH agonists) and/or hormonotherapy (eg, aromatase inhibitors, selective estrogen receptor modulators) seem promising.

Raloxifene has been used in a case of postoophorectomy relapse of BML with good results. In this case,^79^ the patient was postmenopausal. However, in another case, a patient was treated with raloxifene combined with GnRH agonist leuprolide with no improvement in disease control (no menopause status information was provided).^78^ Raloxifene has been shown to induce apoptosis and to reduce cell proliferation in postmenopausal women with leiomyomas, but in premenopausal women it has had mixed effects.^80^ It should, then, be considered for the treatment of BML in postmenopausal women. Tamoxifen, on the other hand, seems to have a deleterious effect by promoting uterine estrogen agonism, and even promoting the growth of metastasizing leiomyomata.^81^,^91^ A review by Lewis et al.^78^ revealed a negative or nonbeneficial impact of tamoxifen in the majority of cases of BML treated with tamoxifen.

Direct hormonal blockade with progesterone has also been tried, but apparently with varying results.^81^ Progesterone suppresses the hypothalamic–pituitary–gonadal axis and hampers the production of estrogen by the ovaries. Moreover, progesterone also increases conversion of estradiol to estrone in endometrial tissue^82^ and decreases intracellular aromatase activity by up to 30%.^83^ Notwithstanding its anti-estrogenic effects, progesterone seems to both trigger the production of the anti-apoptotic Bcl-2 and of the proapoptotic TNF-α.^84^

Hormone therapy coupled with GnRH agonism has yielded positive results in patients with BML.^78^ GnRH agonists not only suppress the ovarian steroidogenesis but also inhibit aromatase.^85^ A randomized, controlled clinical trial comparing the effects of aromatase inhibitor letrozole and GnRH agonist triptorelin on uterine leiomyoma volume showed a volume reduction with both therapies, with a slighter advantage of letrozole over triptorelin^86^. The women in this study were premenopausal. The authors advocate the use of letrozole especially in the context of short-term management in patients who wish to avoid surgical intervention, due to the benefits of rapid onset of action and avoiding at the same time the initial GnRH flare. The use of aromatase inhibitors might be justified by the fact that leiomyomata tend to overexpress aromatase.^78^ However, it must be considered that there might be a wide variation in drug metabolism and drug effects for patients taking aromatase inhibitors, therefore patients should have their own individualized treatment.^78,87^ As is the case with breast cancer patients, sex hormones levels may need to be regularly monitored.^78^

The gold standard for IVL treatment is surgical castration with hysterectomy and bilateral annexectomy, associated with complete resection of intravenous and intracardiac tumors.^7,22,28,29^ Incomplete resections correlate with a higher risk of recurrence, so all stages of the disease should undergo surgery.^21,26^ The importance of surgical castration relies on the presence of hormonal receptors in IVL tumors’ uterine tissue.^26^ Ma et al.^21^ report a series of 76 patients treated for IVL where no patient with IVL grade 2 or more (all of these patients had both ovaries removed) recurred, meaning that removal of both ovaries is essential for prevention of recurrence.^21^ On the other hand, recurrence occurred in four out of seven patients in the IVL grade 1 group who had fertility-sparing surgery.^21^ Surgery of IVL tumors stages 3 and 4 requires extracorporeal circulation.^21,23^ The intravenous tumor is commonly described as an intravenous floating worm with its base adhered to the venous wall in the lower pelvis.^26^ A tumoral stripping technique is managed via the abdomen.^88^ When there is cardiac involvement, a sternotomy with direct cardiac approach is recommended together with the abdominal surgery in a two-staged procedure.^23^ When complete resection is not possible, hormone therapy is an alternative.^26^ Partial resection followed by hormone therapy seems to not modify the risk of recurrence.^22^ Ma et al.^21^ propose cytoreductive preoperative GnRH agonists for improving resectability, and hormone therapy for 6 months postoperatively for recurrence prevention.^21^ Declas and Lucot^26^ propose hormone therapy instead of surgical castration for very young patients, and aromatase inhibitors for very old patients who either refuse annexectomy, or for whom surgery is, for some reason, not possible.

Many LPD cases have an indolent evolution, and many are reported to disappear spontaneously, after menopause, in the postpartum period or when the patient stops taking the contraceptive pill.^6,29^ These findings corroborate the hypothesis of a hyperestrogenism state causing LPD in fertile women. There is no standardized treatment for LPD. Declas and Lucot^26^ have proposed an algorithm for the treatment of LPD. In summary, if the patient is symptomatic, surgical and/or hormone therapy must be administered. Hormone therapy as a form of treatment in fertile women is especially appropriate, because there may be a desire to start a family. For asymptomatic patients, exploratory laparoscopy plus sampling of nodules is suggested. We must know that laparoscopy is a risk considering the hypothesis that seeding of leiomyoma cells is probably a major risk factor for the development of LPD. Biopsy should always be done when attainable.

Follow-up of BML, IVL, and LPD should be long-term and once or twice a year, with a CT scan.^10,11^ In the case of LPD, follow-up is especially important in the first year due to a higher risk of degenerescence into leiomyosarcoma.^6,38,81,89^ This transformation seems to occur more often in patients without an evident hormonal terrain (eg, without a previous history of fibroma or with a hormone receptor non-expressing tumor), for whom hormone therapy might be less effective.^46^ In the case of HLRCC syndrome, given that 6.7% of renal cell carcinoma cases appear before the age of 20, and that the youngest case known is that of a 10-year-old infant, assessments should be started by the age of 8-10, including the search for mutations.^47^ Radiological follow-up is recommended with an annual MRI scan.^90^ Recurrence of IVL happens with a rate of 22.2%^22^ to 30%.^6,7,29^ The rate goes down to 7.6% if resection is complete.^21^ The main risk factors for recurrence of IVL are young age, large initial size of the tumor, incomplete resection without postoperative hormone therapy, and sparing the uterus and the ovaries.^21,22^ Recurrence may occur many years after surgery,^26^ either as IVL, or BML, or even LPD.^27^ If recurrence happens, repeating a radical resection with free margins improves the patient’s prognosis significantly.^27^ An MRI scan is suggested for the first 3 to 6 months postcomplete resection of IVL, with follow-up with MRI scans every 2 to 5 years, according to the severity and/or staging of the disease.^27^

Cases 1, 2, and 3 were premenopausal at the time of diagnosis, which is consistent with cases reported in the literature. Women diagnosed with BML tend to be pre- or perimenopausal.^13,16,50,77^ In Case 1, both pulmonary and cutaneous metastasizing leiomyomatosis were found. Cutaneous and uterine leiomyomatosis is associated to renal cell cancer in HLRCC syndrome. HLRCC syndrome is associated to a germline mutation in fumarate hydratase, a tumor suppressor gene. Although the patient from Case 1 was never diagnosed with renal cell cancer and had no family history of cancer or leiomyomatosis, the presence of multiple cutaneous and uterine leiomyomas (MCUL) and the lack of expression of fumarate hydratase in the lung tissue we had access to points to a possible HLRCC syndrome diagnosis. The patient has thus already been referred to our Family Risk Clinic. Given that this seems to be a new case in the family, a de novo mutation might have caused the patient to develop MCUL. It is worth noting that in Case 2 the patient was completely asymptomatic, and yet in Case 1 BML was discovered after a period of cough associated to an upper respiratory infection. In Case 3 the patient had a past history of childhood asthma as a confounding factor for her symptoms of dyspnea and wheezing. This is probably related to her left lung hilar mass compressing the left main bronchus wall. We recommend screening for pulmonary BML in hysterectomized women with a history of chronic cough or frequent respiratory infections. Screening for BML in the lungs and other organs should also be offered to patients with non-specific symptoms. Genetic screening in women undergoing surgery due to uterine leiomyomatosis, namely looking for deletions in chromosomes 1p, 19q and 22q, is not recommended, given the rarity of BML.^49^

It is interesting to note that in the first two cases the span of time between hysterectomy and diagnosis of BML, 16 years (Case 1) and 22 (Case 2) years, was similar or above the times of 3 years,^13^ 8.8 years (mean),^16^ 18.5 years,^50^ and 16 years^77^ described in the literature, while in Case 3 the patient developed symptoms just 4 years after hysterectomy. Also, the first three patients were slightly younger, with a diagnosis of BML at the age of 39 (Case 1), 44 (Case 3), and 45 (Case 2), than what was reported in the literature (47.3 years (mean),^16^ 47.5 years,^77^ 51 years,^13^ 55 years^50^). Moreover, in Case 1, not only was the patient diagnosed at a younger age, but the disease itself was more aggressive, with progression first in the size and number of lung nodules, then progression to the skin. Disease aggressiveness in Case 1 was apparently independent of surgical castration. This aggressiveness might be related to the possible HLRCC syndrome diagnosis and the deficient expression of fumarate hydratase. Benign metastasizing leiomyomatosis in Case 2 was much less aggressive, with disease stability achieved with GnRH agonist goserelin monotherapy despite the prolonged fertility period. This patient was still premenopausal at the age of 56 and has a family history of late menopause. Benign metastasizing leiomyomatosis in Case 3 seemed to respond well, first to double hormonal blockade of goserelin + letrozole, and later, after surgical castration, to isolated letrozole.

Cases 4 and 5 point to the need to regularly screen for recurrence of disease in IVL and LPD patients. This is especially true for IVL, since it has a high risk of recurrence.

It is important to consider the hormonal status in patients with BML. Patients’ hormonal status should be determined because it can direct the choice for hormonal blockade. All our three cases of BML (Cases 1, 2, and 3) occurred in premenopausal patients, while IVL in Case 4 and LPD in Case 5 both occurred in postmenopausal patients. BML, IVL, and LPD seem to be more frequent in fertile women. Also, in the case of fertile patients, especially those with an indication for surgery, the issues of pregnancy planning and fertility preservation should be addressed and discussed with each patient individually. Benign metastasizing leiomyomatosis, IVL, and LPD should be taken into account in patients submitted to hysterectomy due to uterine leiomyomatosis who develop chronic cough, frequent respiratory infections, abdominal discomfort, right heart failure, or non-specific symptoms. Also, it is of the utmost importance to use a collecting bag when performing surgery on leiomyomatosis, primary or secondary, to avoid the potential spreading of leiomyomatosis.

## Conclusion

Benign metastasizing leiomyomatosis is a rare disease that occurs most often in women with a previous history of uterine leiomyomatosis. Given its rarity, there are no official guidelines for treatment. There seems to be some genetic association between chromosomal deletions 1p, 19q, and 22q in uterine leiomyomata and BML nodules. BML, IVL and LPD are rare but potentially threatening variants of extra-uterine leiomyomatosis. All three conditions should be taken into account in hysterectomized patients with chronic cough, frequent respiratory infections, abdominal discomfort, right heart failure, or non-specific symptoms. If cutaneous nodules are also present, HLRCC should be suspected. Treatment should be individualized according to each patient’s hormonal status and desires.

## Data Availability

The data underlying this article will be shared on reasonable request to the corresponding author.
